# Statistical Issues in Farmworker Studies

**DOI:** 10.1289/ehp.114-1764171

**Published:** 2006-12

**Authors:** David T. Mage, Lance A. Wallace, Mel Kollander, Wayne R. Ott

**Affiliations:** Temple University (retired), Newark, Delaware, E-mail: magedonner@aol.com; U.S. Environmental Protection Agency (retired), Reston, Virginia; Temple University (retired), Alexandria, Virginia; Stanford University, Stanford, California

Barr et al. (2006) surveyed statistical issues related to farmworker exposure studies. However, they made several factual and conceptual errors that need to be called to the readers’ attention.

First, Barr et al. (2006) claimed incorrectly that “representativeness” is optional and not a necessary condition for a well-designed investigation. For convenience samples,

[T]he results only pertain to the sample itself, and should not be used to make quantitative statements about any population – including the population from which the sample was selected.” [U.S. Environmental Protection Agency (EPA) 2003]

Barr et al. (2006) stated that “because responses from convenience samples are likely to be better than that for a representative sample, they may actually be more ‘representative.’” The fallacy of this statement is shown by a hypothetical CNN call-in response to a question from 100% of its viewers that perfectly represents all CNN viewers. In this illustrative example, the 100% response would not represent the entire population of the United States as well as a probability-based survey of the U.S. population that included non-CNN viewers that achieved an 80% response rate.

In their article, Barr et al. (2006) claimed that “perfectly random sampling across all relevant factors is therefore almost universally impractical.” Acquavella et al. (2004) monitored a probability sample of pesticide applicators; U.S. EPA provided several TEAM (Total Exposure Assessment Methodology) studies using a scientific probability design (Thomas 1993; Wallace 1991; Wallace et al. 1987), as did the World Health Organization, U.S. EPA, and Harvard University for the government of Kuwait during the 1991 oil fires (Mage DT, Wallace LA, Kollander M, personal communication). The Centers for Disease Control and Prevention’s (CDC) National Health and Nutrition Examination Survey study (CDC 2003) is another excellent example of proper probability-based sample selection.

According to Barr et al. (2006), it is possible to identify and “sample known or anticipated ‘hot spots’ of [pesticide] exposure.” There are only two categories of applicators expected to be at high risk of a high pesticide exposure event: the inexperienced applicators who are still learning how to apply pesticides safely, and those applicators who do not follow the mandatory manufacturer’s label requirements in violation of federal law (Mage et al. 2002). Whereas the former cohort might be identified by a screening question about prior numbers of applications, there is no certain way to identify the latter group, who will likely not admit to taking shortcuts or refusing to use required personal protective equipment, because they might be incriminating themselves. Finally, such an applicator may succumb to the Hawthorne effect [not mentioned by Barr et al. (2006) as a caveat], defined by Last (1988) as “the effect of being under study upon the persons being studied.”

Barr et al. (2006) claimed that “some form of convenience sampling is typically adopted in practice.” Unfortunately, this claim is true; some of these authors did use convenience sampling in previous studies (Curwin et al. 2002, 2005) in which subjects were recruited by “word of mouth.” A friend or neighbor recruited by an enrolled subject might not be “an independent sample” if he or she has some similar characteristics (e.g., crops grown, acreage, age, race, education, sex) as the recruiter. This haphazard practice of using volunteers for convenience, or even subjects based on expert choice (Hoppin et al. 2006), limits the validity of the study, as theoretical confidence intervals and significance *p*-values become meaningless.

The weakness of all nonprobability sampling is its subjectivity that precludes the development of a theoretical framework for it. (Kalton 1983)

Finally, as former U.S. EPA scientists who pioneered agency exposure science, we are disappointed that this article was cleared for publication by the U.S. EPA because it is not in accordance with U.S. EPA (and other agency) requirements to follow the Office of Management and Budget’s (OMB) data collection policies (OMB 2006) that require “selecting samples using generally accepted statistical methods (e.g., probabilistic methods that can provide estimates of sampling error).” The U.S. EPA (2003) stated:

Probability sampling must be used at each stage of respondent selection. You may encounter difficulties in clearing the survey through OMB if you do not insist that probability selection methods be used.

Recent samples of high-risk subpopulations and their exposures to particles were undertaken by the U.S. EPA using doctor-identified subjects, and these were therefore not probability-based samples. The OMB allowed these studies but required that a statement be made in all resulting publications that the results could be applied only to the participants, even if chosen in this case by expert judgment, and must not be extrapolated to larger populations. We believe a similar statement should be made in all publications of studies using alternatives to probability-based sampling.

In summary, Barr et al. (2006) attempted to review survey design practices, but they do not seem to understand that the convenience samples they advocate apply only to the subjects selected and not to the larger populations from which they are taken.
